# Demographic and Clinical Features of Small-for-Gestational-Age Infants Born to Mothers With Gestational Diabetes Mellitus

**DOI:** 10.3389/fped.2021.741793

**Published:** 2021-10-01

**Authors:** Juncao Chen, Huimin Xiao, Yong Yang, Yaping Tang, Xiaoqi Yang, Zhe Zhang, Weineng Lu, Jie Yao, Longguang Huang, Xiaoping Liu, Wei Zhou

**Affiliations:** ^1^Department of Neonatology, Guangzhou Women and Children's Medical Center, Guangzhou Medical University, Guangzhou, China; ^2^Department of Neonatology, Dongguan Houjie Hospital, Guangdong Medical University, Dongguan, China; ^3^Department of Neonatology, Dongguan City Maternal and Child Health Hospital, Southern Medical University, Dongguan, China; ^4^Institute of Pediatrics, Guangzhou Women and Children's Medical Centre, Guangzhou Medical University, Guangzhou, China; ^5^Department of Hematology, Guangzhou Women and Children's Medical Center, Guangzhou Medical University, Guangzhou, China

**Keywords:** small-for-gestational-age, infants, gestational diabetes mellitus, clinical characteristic, risk factors, catch-up growth

## Abstract

We studied the demographic and clinical characteristic, risk factors, outcomes of full-term small-for-gestational-age (SGA) infants born to mothers with gestational diabetes mellitus (GDM) in China. A retrospective case-control study that included 1981 SGA infants was conducted; the demographic and clinical data between SGA infants born to mothers with and without GDM were compared. Of 383 SGA infants born to mothers with GDM, 221 (57.7%) were female, and the incidence of these infants was 1 in 155 live births. The risk of SGA siblings (RR, 1.88; 95% CI, [1.23–2.86]), low 1- and 5-min Apgar scores (RR,2.04 and 4.21; 95%CI [1.05–4.00] and [1.05–16.89], respectively), early thrombocytopenia (RR, 3.39; 95%CI, [1.33–8.64]), hypoglycemia(RR, 2.49; 95%CI, [1.55–3.98]), and hypoxic-ischemic encephalopathy (RR,5.61; 95%CI, [1.25–25.18]) were increased in SGA infants born to mothers with GDM compared to SGA infants born to mothers without GDM. SGA girls born to mothers with GDM had a significantly higher ratio of catch-up growth (CUG) (RR, 1.73; 95%CI, [1.18–2.54]) in the first year of life. These results show that genetic factors may be one of the etiologies of SGA infants born to mothers with GDM; and these infants have more adverse perinatal outcomes compared to SGA infants born to mothers without GDM. SGA girls born to mothers with GDM had accelerated CUG in the first year of life.

## Introduction

Gestational diabetes mellitus (GDM) is a major public health problem for women in China and worldwide (1). Fetuses receive increased amounts of glucose from mothers with GDM during pregnancy, which promotes insulin secretion and increases fetal growth. However, an epidemiological study found that 7% of infants whose mothers had GDM were small- for-gestational-age (SGA) in the United States (2). The term of SGA refers to infants with birth weights (BW) below the 10th percentile for gestational age (3). To date, there have been no studies in China on the incidence of SGA infants born to mothers with GDM. The causes of SGA include genetic and environmental factors, but the cause of SGA infants born to mothers with GDM is unknown.

GDM and SGA are associated with delayed development and poor growth of the fetuses. Epidemiological studies showed that infants born to mothers with GDM had a higher rate of adverse perinatal outcomes (e.g., asphyxia, hypoglycemia, and birth defects) than those in infants born to mothers without GDM; but the clinical characteristics of SGA infants born to mothers with GDM are poorly understood (4, 5). Therefore, there is a clear need for further investigation of the clinical characteristics of SGA infants born to mothers with GDM.

Multiple epidemiological studies have shown that SGA infants and infants born to mothers with GDM share common long-term complications (cardiovascular disease, type 2 diabetes) in adulthood and biological pathways leading to these long-term complications; studies also showed that faster post-natal growth in these infants appeared to be an important determinant of cardiovascular disease and type 2 diabetes (6–10). This pattern of growth, or “catch-up growth (CUG)”, was defined as the faster post-natal growth in response to recovery from undernutrition *in-utero*. Thus, we evaluated the outcomes of SGA infants born to mothers with GDM by measuring weight gain and the ratio of CUG in the first year of life.

In this study, we sought to investigate the demographic and clinical characteristics, risk factors, and outcomes of SGA infants born to mothers with GDM in China.

## Methods

This study was a population-based, retrospective case-control study; written informed consent was provided by all parents. This study was approved by the research ethical committee of Guangzhou Women and Children's Medical Center, Dongguan Houjie hospital and Dongguan city maternal and child health hospital; all procedures performed in studies involving human participants were in accordance with the relevant guidelines and regulations.

### Study Design and Participants

We performed a retrospective case-control study on demographic and clinical features of SGA infants born to mothers with GDM, and infants delivered in Guangzhou Women and Children's Medical Center, Dongguan Houjie hospital and Dongguan city maternal and child health hospital between January 1, 2008 and October 31, 2018. All mothers giving births to SGA infants who underwent regular obstetric examination in hospitals and had undergone a 75 g oral glucose tolerance test (OGTT) following the standard protocol at 24–28 weeks of gestation at the Department of Obstetrics were included. GDM was diagnosed when any one value reaches or exceeds 5.1 mmol/L at 0 h, 10.0 mmol/L at 1 h, or 8.5 mmol/L at 2 h (11).

All included mother/infant pairs met the following inclusion criteria: (1) Chinese nationality and gestational age ≥37 weeks (gestational age was assessed on the basis of the last menstruation and identified by early ultrasound pregnancy prior to 20 weeks' gestation); preterm were excluded from analysis because Chinese children growth trajectories are inapplicable to these infants; (2) No chromosomal aberrations and genetic syndromes in infants; (3) mothers between 20 and 40 years of age and having no disorders known to affect glucose metabolism including diabetes, polycystic ovarian syndrome and uncontrolled thyroid during pregnancy; and (4) mothers/infant pairs with complete maternal (delivery data, complication during pregnancy) and neonatal data (birth data, in-hospital outcomes). Mothers/infant pairs were excluded if they did not meet all of these inclusion criteria. Only one birth for mother was included in our study.

All infants included in our study were divided into two groups. Group 1 (case group) included SGA infants born to mothers with GDM. Group 2 (control group) included SGA infants born to mothers without GDM. We performed 1:4 (case group: control group) matching according to neonatal gestational age (difference was ≤ 3 days), gender, birth weight range (i.e., >2,500, 2,000–2,500, and <2,000g) and maternal age (difference was ≤ 3 years), place of residence. The definition of SGA was birth weights ≤ 10th percentile for the gestational age based upon defined standards for males and females according to Chinese Neonatal Network (12).

### Data Collection and Definition

All mother/infant pairs underwent structured medical examinations and physical examinations. Data on mothers and neonates were obtained from medical records. The weight and length/height of mother/infant pairs was measured by trained nurses using standard anthropometric methods, pre-pregnancy weight obtained according to maternal self-report. Body mass index (BMI) was calculated by dividing weight in kilograms by the square of height in meters. Early thrombocytopenia was defined as a platelet count of <1,501 × 0^9^/l in the first 72 h of life. Hypoglycemia was defined as blood glucose <35 mg/dl or plasma glucose <40 mg/dl. The diagnostic criteria for hypoxic-ischemic encephalopathy (HIE) were: (1) evidence of clinical encephalopathy in the first 12 h of life; (2) history of perinatal asphyxia; (3) Apgar score ≤ 5 at 5 min; and with at least one of the following: (a) continued need for positive pressure ventilation for 10 min or history of cardiopulmonary resuscitation at birth; and/or b) pH ≤ 7.00 in arterial cord blood or other sample or base deficit ≤ 12 mmol/l. HIE was classified according to modified Sarnat criteria. The grade for ICH was classified according to Papile criteria. Late-onset sepsis, occurring after 72 h from birth, was defined as positive blood culture. Necrotizing enterocolitis (NEC) was diagnosed according to Bell criteria. Neonatal respiratory distress syndrome (NRDS) was diagnosed by (1) evidence of respiratory failure, (2) administration of exogenous pulmonary surfactant (3) radiographic evidence. The criteria of maternal diseases listed in Table 1. All infants were invited for follow-up measurements of weight at 12 months of age, and CUG was defined as a gain in the SD score for weight from birth to 1 year of age that was >0.67 standard deviations (SD) scores (10). Age- and gender-adjusted SD scores for weight were calculated according to the growth reference standard for Chinese children under 7 years of age (12).

**Table 1:** Maternal characteristics of women with and without gestational diabetes mellitus (*N* = 1,981).

	SGA mothers with GDM^*^ (*n* = 383)	**SGA mothers without GDM (*n* = 1,598)**	**RR (95% CI)**	***p* Value**
Age, mean (SD), years	31.2 (4.7)	29.8 (4.3)		<0.01
Race, No. (%)			1.16 [0.66–2.04]	0.61
Han nationality	368 (96.1)	1,526 (95.5)		
other nationalities	15 (3.9)	72 (4.5)		
Residence, No. (%)			0.87 [0.61–1.24]	0.44
Rural	41 (10.7)	194 (12.1)		
Urban	342 (89.3)	1,404 (87.9)		
Height, mean (SD), cm,	157.7 (4.7)	158.2 (5.3)		0.09
Gravidity, mean (SD)	2.0 (1.3)	1.6 (0.9)		<0.01
Parity (primiparous), No. (%)	256 (66.8)	1,056 (66.1)	1.04 [0.82–1.31]	0.78
Pre-pregnancy weight, mean (SD), kg	51.6 (7.8)	51.1 (7.0)		0.01
Pre-pregnancy BMI, mean (SD), kg/m^2^	20.8 (2.8)	20.0 (2.9)		<0.01
Pre-partum weight, mean (SD), kg	63.0 (8.3)	62.5 (7.9)		0.38
Pre-partum BMI,^†^ mean (SD), kg/m^2^	25.1 (3.7)	24.7 (4.1)		0.11
Increased weight during pregnancy, mean (SD), kg	11.8 (4.6)	12.8 (3.9)		<0.01
Complications during pregnancy				
Anemia, No, (%)	29 (7.6)	155 (9.7)	0.76 [0.51–1.15]	0.20
Abnormal placenta,^‡^ No, (%)	7 (1.8)	26 (1.6)	1.13 [0.49–2.61]	0.78
Chorioamnionitis, No, (%)	4 (1.0)	24 (1.5)	0.69 [0.24–2.01]	0.50
Drug or illicit use, No, (%)	1 (0.3)	3 (0.2)	1.39 [0.14–13.42]	0.77
Tobacco use, No, (%)	2 (0.5)	13 (0.8)	0.64 [0.14–2.85]	0.56
Hypothyroidism, No, (%)	11 (2.9)	56 (3.5)	0.81 [0.42–1.57]	0.54
ICP, No, (%)	2 (0.5)	20 (1.3)	0.41 [0.10–1.78]	0.22
Intrauterine distress, No, (%)	9 (2.3)	34 (2.1)	1.11 [0.53–2.33]	0.79
Maternal heart diseases,^§^ No, (%)	1 (0.3)	5 (0.3)	0.83 [0.10–7.16]	0.89
Oligohydramnios,^||^ No, (%)	10 (2.6)	129 (8.1)	0.31 [0.16–0.59]	<0.01
Pregnancy-induced hypertension, No, (%)	22 (5.7)	65 (4.1)	1.44 [0.88–2.36]	0.15
Preeclampsia No, (%)	20 (5.2)	92 (5.8)	0.90 [0.55–1.48]	0.68
Placenta previa, No, (%)	7 (1.8)	31 (1.9)	0.94 [0.41–2.15]	0.89
Placental abruption, No, (%)	4 (1.0)	30 (1.9)	0.55 [0.19–1.58]	0.26
Placental insufficiency,^¶^ No, (%)	23 (6.0)	152 (9.5)	0.61 [0.39–0.96]	0.03
Renal diseases,^#^ No, (%)	1 (0.3)	8 (0.5)	0.52 [0.07–4.17]	0.53
Single umbilical artery, No, (%)	2 (0.5)	4 (0.3)	2.01 [0.38–11.46]	0.38
Uterine malformation,^**^ No, (%)	2 (0.5)	11 (0.7)	0.76 [0.17–3.43]	0.72
Delivery				
Breech, No, (%)	16 (4.2)	52 (3.3)	1.30 [0.73–2.30]	0.37
Vaginal, No, (%)	307 (80.2)	1,022 (64.0)	2.28 [1.74–2.99]	<0.01
Failed forceps, No, (%)	10 (2.6)	45 (2.8)	0.93 [0.46–1.85]	0.83
Cesarean section, No, (%)	50 (13.0)	461 (28.8)	0.37 [0.27–0.51]	<0.01
Clinical Characteristics of SGA mothers with GDM				
Diet treatment for GDM, No, (%)	380 (99.2)	NA		NA
Drug treatment for GDM, No, (%)	3 (0.8)	NA		NA
Recurrent GDM, No, (%)	18 (4.7)	NA		NA

*BMI, body mass index; NA, not applicable; ICP, intrahepatic cholestasis of pregnancy; GDM, gestational diabetes mellitus; RR, relative risk; SD, standard deviations; SGA, small for gestational age;*.

* *, GDM diagnosed by IADPSG criteria published in 2010*.

† *, BMI, weight (kg)/length (m)^2^*.

‡ *, abnormal placenta contain velamentous placenta and battledore placenta*.

§ *, Maternal heart diseases contain congenital heart disease, cardiomyopathy, pericarditis and heart failure*.

|| *, Oligohydramnios can be defined as amniotic fluid volume <5% for gestational age, AFI <5 cm or maximal deepest pocket <2 cm*.

¶ *, Placental insufficiency can be defined as 36-week cerebroplacental ratio <5th centile, umbilical artery blood gas analysis pH <7.15, or placental weight <10th centile*.

# *, Renal diseases contain nephrotic syndrome, lupus nephritis, chronic nephritis and renal failure*.

** *, Uterine malformation contain uterus hypoplasia, monocular uterus, double uterus, biangular uterus, mediastinal uterus and arch uterus*.

### Sample Size and Statistical Analysis

Sample size calculation was performed using PASS 15. With 90% power and the assumption of relative risk = 2.0, we calculated that 256 SGA infants born to mothers with GDM were needed; four comparison individuals per case, 1024 SGA infants born to mothers without GDM were needed. Statistical calculations were performed by using SPSS version 22. For the statistical analysis, *t-*tests were used for continuous variables that were described with the means ± standard deviations (*x*^−^ ± *s*). χ^2^ tests were used for categorical variables that were described with frequencies and percentages. Multivariate binary Logistic regression was used to determine risk factors of SGA infants born to mothers with GDM. Pearson's correlation coefficients were conducted to determine the association between maternal factors (weight gain during pregnancy, BMI in the intrapartum period and BMI in the pre-pregnancy period) and birth weight. The significance level was set at *p* < 0.05.

## Results

### Population

Of 160,474 infants that were screened in our study from 2008 to 2018, 16,059 were SGA infants (10.0%, SGA was defined as birth weights ≤10th percentile for the gestational age based upon defined standards for males and females), 15,726 infants born to mothers with GDM(9.8%). Of 160,474 infants that were screened, 1,034 were SGA infants born to mothers with GDM (0.6%), which yielded an incidence for full-term SGA infants born to mothers with GDM of approximately 1 in 155 live births. Of 1,034 SGA infants born to mothers with GDM, 808 singleton births, 220 twins births, and six triplets.

**Figure 1:**
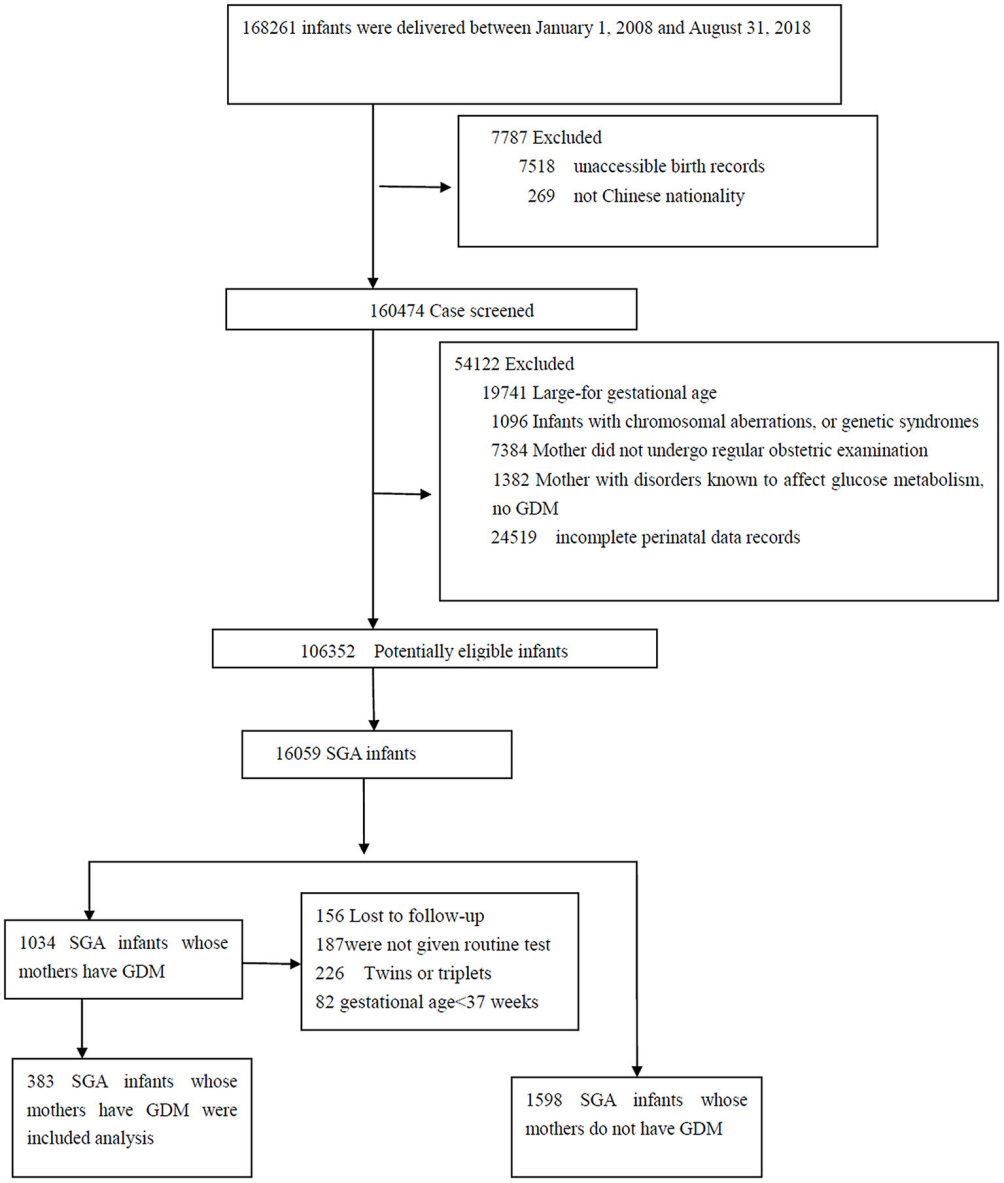
Flow diagram of study population.

The final sample consisted of 1,981 SGA infants: 383 full-term SGA infants born to mothers with GDM, 1,598 full-term SGA infants born to mothers without GDM (Figure 1). Groups did not differ significantly in sex, birth weight or most demographic characteristics (all *P* >0.05) (Tables 1, 2).

**Table 2:** Clinical characteristics of small-for-gestational-age infants born to mothers with and without gestational diabetes mellitus (*N* = 1,981).

	**SGA infants born to mothers with GDM (*n* = 383)**	**SGA infants born to mothers without GDM (*n* = 1,598)**	**RR (95%CI)**	***p* Value**
Gestational age, mean (SD), weeks	38.4 (1.0)	38.5 (1.1)		0.04
Sex (boys/girls)	172:221	647:951	1.14 [0.92–−1.43]	0.24
Birth weight (BW), mean (SD), g	2,418.2 (211.6)	2,397.0 (206.1)		0.08
>2,500 g, No, (%)	71 (18.5)	262 (16.4)	1.16 [0.87–1.55]	0.31
2,000–2,500 g, No, (%)	299 (78.1)	1,278 (80.0)	0.89 [0.68–1.17]	0.41
<2,000 g, No, (%)	13 (3.4)	58 (3.6)	0.93 [0.51–1.72]	0.82
Birth length, mean (SD), cm	46.5 (1.8)	46.3 (2.1)		0.14
BMI,^*^ kg/m^2^, mean (SD)	11.0 (0.9)	11.0 (1.4)		0.47
Head circumference, mean (SD), cm	31.9 (1.2)	31.8 (1.2)		0.95
Ponderal index^†^			0.91 [0.70–1.17]	0.46
>2.2, No, (%)	286 (74.7)	1,222 (76.5)		
<2.2, No, (%)	97 (25.3)	376 (23.5)		
Siblings who were SGA, No, (%)	43 (33.9)	116 (21.4)	1.88 [1.23–2.86]	<0.01
Apgar score				
1 min, Low (≤ 7), No, (%)	13 (3.4)	27 (1.7)	2.04 [1.05–4.00]	0.03
1 min, Low (≤ 3), No, (%)	4 (1.0)	3 (0.2)	5.61 [1.25–25.18]	0.01
5 min, Low (≤ 7), No, (%)	4 (1.0)	4 (0.3)	4.21 [1.05–16.89]	0.03
10 min, Low (≤ 7), No, (%)	1 (0.3)	2 (0.1)	2.09 [0.19–23.10]	0.54
**Perinatal complications**				
Early thrombocytopenia, No, (%)	8 (2.1)	10 (0.6)	3.39 [1.33–8.64]	<0.01
Gastrointestinal hemorrhage, No, (%)	9(2.3)	19 (1.2)	2.00 [0.90–4.46]	0.08
HIE,^‡^ No, (%)	4 (1.0)	3 (0.2)	5.61 [1.25–25.18]	0.01
Hypoglycemia, No, (%)	29 (7.6)	51 (3.2)	2.49 [1.55–3.98]	<0.01
ICH				
ICH (I and II), No, (%)	14 (3.7)	36 (2.2)	1.65 [0.88–3.01]	0.12
ICH (III and IV), No, (%)	0	4 (0.3)	1.00 [1.00–1.005]	0.33
Late-onset sepsis, No, (%)	6 (1.6)	17 (1.1)	1.48 [0.58–3.78]	0.41
Neonatal hypoglycemia encephalopathy, No, (%)	2 (0.5)	2 (0.1)	4.19 [0.59–29.83]	0.12
NEC, No, (%)	3 (0.8)	9 (0.6)	1.39 [0.38–5.17]	0.62
NRDS, No, (%)	2 (0.5)	3 (0.2)	2.79 [0.47–16.76]	0.24
Obstetric trauma, No, (%)	8 (2.1)	11 (0.7)	3.08 [1.23–7.71]	0.01
Pulmonary hemorrhage, No, (%)	3 (0.8)	2 (0.1)	6.38 [1.06–38.34]	0.02
Symptomatic polycythemia, No, (%)	1 (0.3)	10 (0.6)	0.42 [0.05–3.26]	0.39
**Birth defects**				
Cleft palate, No, (%)	0	4 (0.3)	1.003 [1–1.005]	0.33
Hypospadias, No, (%)	2 (0.5)	10 (0.6)	0.83 [0.18–3.82]	0.81
Polydactyly, No, %	1 (0.3)	10 (0.6)	0.42 [0.05–3.26]	0.39
Ventricular septal defect, No, (%)	3 (0.8)	17 (1.1)	0.73 [0.21–2.52]	0.62

*BMI, body mass index; CI, confidence interval; GDM, gestational diabetes mellitus; HIE, hypoxic-ischemic encephalopathy; ICH, Intracranial hemorrhage; NEC: Necrotizing enterocolitis; NRDS, neonatal respiratory distress syndrome; NA, not applicable; RR, relative risk; SD, standard deviations; SGA, small for gestational age*.

* *, BMI, weight (kg)/length (m)^2^*.

† *, Ponderal index, ([birth weight (g)]/ [crown heel length (cm)]^3^) ×100*.

‡ *, SGA infants born to mothers with GDM: 1 mild HIE, 1 moderate HIE, 2 severe HIE; SGA infants born to mothers without GDM: 0 mild HIE, 1 moderate HIE, 2 severe HIE*.

A total of 383 eligible SGA mothers with GDM at a mean age of 31.2 (SD 4.7) years were included, 380 (99.2%) women with GDM were given dietary therapy. Significant differences were found in pre-pregnancy weight (51.67 ± 0.8 vs. 51.17 ± 0.0 kg), pre-pregnancy BMI (20.82 ± 0.8 vs 0.202 ± 0.9), increased weight during pregnancy (11.84 ± 0.6 vs. 12.83 ± 0.9 kg), and the incidence of vaginal delivery (80.2 vs. 64.0%; relative risk [RR] 2.28; 95% confidence interval [CI] [1.74–2.99]), cesarean section (RR, 0.37; 95%CI, [0.27–0.51]) between SGA mothers with and without GDM groups. Additional population characteristics were available in Table 1.

### The Comparison of Clinical Characteristics in SGA Infants Born to Mothers With GDM to These Mothers Without GDM

Table 2 showed the clinical characteristics of SGA infants born to mothers with and without GDM. 286 (74.7%) SGA infants born to mothers with GDM had a high ponderal index (PI > 2.2), and could be classified as symmetric SGA, but no significant difference for PI was found between the 2 groups.

The rate of SGA siblings in the SGA infants born to mothers with GDM was higher than that in the SGA infants born to mothers without GDM (33.9 vs.21.4%; RR 1.88; 95% CI [1.23–2.86]). The risk of low 1- and 5-min Apgar scores (RR,2.04 and 4.21; 95%CI, [1.05–4.00] and [1.05–16.89], respectively), early thrombocytopenia (RR, 3.39; 95%CI, [1.33–8.64]), HIE (RR,5.61; 95%CI, [1.25–25.18]), hypoglycemia (RR,2.49; 95%CI, [1.55–3.98]), obstetric trauma (RR,3.08; 95% CI, [1.23–7.71]), and pulmonary hemorrhage (RR,6.38; 95%CI, [1.06–38.34]) were increased in SGA infants born to mothers with GDM compared to SGA infants born to mothers without GDM.

### Infant Outcomes

The outcomes of the infants were summarized in Table 3. Follow-up data were obtained for 334 SGA infants born to mothers with GDM and 1,364 SGA infants born to mothers without GDM in the first year of life. The rate of CUG (24.9 vs. 16.0%; RR, 1.73; 95%CI, 1.18–2.54) and weight at 1 year age (87,081 ± 02 vs. 84,961 ± 03 g, *P* < 0.01) were significantly higher in the group of SGA girls born to mothers with GDM than that in the group of SGA girls born to mothers without GDM. All SGA infants with severe perinatal complications were followed for 2 years. In the group of SGA infants born to mothers with GDM group, cerebral palsy occurred in two cases (0.6%), short bowel syndrome occurred in one case with NEC (0.3%), and there was one death (0.3%).

**Table 3:** The follow-up outcomes of SGA infants born to mothers with and without GDM (*N* = 1,698).

	**SGA infants born to mothers with GDM (*N* = 334)**	**SGA infants born to mothers without GDM (*N* = 1,364)**	**RR (95%CI)**	***p* Value**
Maternal characteristic				
Race, No. (%)			0.69 [0.37–1.29]	0.24
Han nationality	322 (96.4)	1,294 (94.9)		
Other nationality	12 (3.6)	70 (5.1)		
Residence, No. (%)			1.11 [0.81–1.53]	0.52
Rural	57 (17.1)	213 (15.6)		
Urban	277 (82.9)	1,151 (84.4)		
Maternal age, mean (SD), g	30.9 (4.6)	29.9 (4.2)		<0.01
≥30 years	181 (54.2)	668 (49.0)	1.23 [0.97–1.57]	0.09
<30 years	153 (45.8)	696 (51.0)		
Parity (primiparous), No. (%)	251 (75.1)	972 (71.3)	1.22 [0.93–1.61]	0.16
Drug or illicit use, No, (%)	0	3 (0.2)		0.39
Tobacco use, No, (%)	0	0	NA	NA
High school graduate, No, (%)	189 (56.6)	839 (61.5)	0.82 [0.64–1.04]	0.10
Neonatal characteristic				
Sex (boys/girls)	138:196	590:774	0.92 [0.73–1.18]	0.52
Gestational age, mean (SD), weeks	38.3 (1.0)	38.2 (0.9)		0.64
Birth weight (BW), mean (SD), g	2,377 (185)	2,355 (173)		0.06
Mode of infant feeding within the first 6 months				
Exclusive breastfeeding, *N*, (%)	154 (46.1)	833 (61.1)	0.55 [0.43-0.69]	<0.01
Non- breastfeeding,^*^N, (%)	180 (53.9)	531 (38.9)		
Weight (girls), mean (SD), g^†^	8,708 (102)	8,496 (103)		<0.01
Weight (boys), mean (SD), g	8,825 (114)	8,832 (136)		0.87
Catch-up growth^‡^, *N*, (%)	75 (22.5)	228 (16.7)	1.44 [1.08–1.94]	0.01
Catch-up growth (girls), *N*, (%)	46 (24.9)	135 (16.0)	1.73 [1.18–2.54]	<0.01
Catch-up growth (boys), *N*, (%)	29 (19.5)	93 (17.8)	1.12 [0.70–1.77]	0.65
Outcomes in the 2 years				
Cerebral palsy, No, %	2 (0.6)	6 (0.4)	1.36 [0.27–6.79]	0.70
Short bowel syndrome, No, %	1 (0.3)	2 (0.1)	2.05 [0.19–22.63]	0.55
Mortality, No, %	1 (0.3)	3 (0.2)	1.36 [0.14–13.14]	0.79

*CI, confidence interval; GDM, gestational diabetes mellitus; NA, not applicable; RR, relative risk; SD, standard deviations; SGA, small for gestational age*.

* *, Includes mixed breast and formula, exclusive formula feeding*.

† *, a gain in SD score for weight from birth to 1 years of age that was greater than 0.67 SD scores was taken to indicate clinically significant catch-up growth*.

### Risk Factors

Pearson's correlation coefficients were conducted to determine the association between maternal factors, results are shown in Table 4. The results illustrated that prepregnancy BMI and prepartum period BMI were correlated with birth weight (r, −0.06 and −0.08; 95%CI, −0.12- −0.01 and −0.16 −0.01; respectively).

**Table 4:** Relationships between birth weight and Weight gain during pregnancy, Prepartum period BMI, Prepregnancy BMI in the study subjects (*N* = 1,981).

	**Pearson correlation coefficient (r)**	**95% CI**	***P*-value**
Prepartum period BMI	−0.08	−0.16–0.01	0.03
Prepregnancy BMI	−0.06	−0.12– −0.01	0.02
Weight gain during pregnancy	0.04	−0.04–0.12	0.28

*BMI, body mass index; CI, confidence interval*.

Multivariate binary logistic regression was used to determine the associations between maternal complications and GDM; all results were listed in Table 5. The risks of oligohydramnios (OR, 3.19; 95%CI, [1.66–6.15])were higher in the mothers without GDM compared to mothers with GDM (*P* < 0.05).

**Table 5:** Binary logistic analysis for the associations between maternal complications and gestational diabetes mellitus among SGA infants (*N* = 1,981).

	OR^*^	**95%CI**	***P* value^†^**
Abnormal placenta	0.92	0.39–2.16	0.85
Anemia	1.27	0.84–1.92	0.27
Chorioamnionitis	1.58	0.54–4.60	0.40
Drug or illicit use	0.97	0.09–9.96	0.98
Hypothyroidism	1.28	0.66–2.48	0.46
ICP	2.51	0.58–10.82	0.22
Maternal heart diseases	1.25	0.14–10.86	0.84
Multiple births	0.79	0.45–1.37	0.39
Oligohydramnios	3.19	1.66–6.15	<0.01
pregnancy-induced hypertension	0.75	0.45–1.23	0.26
Preeclampsia	1.03	0.70–1.51	0.89
Placenta previa	1.12	0.49–2.56	0.80
Placental abruption	2.03	0.71–5.83	0.19
Placental insufficiency	1.59	0.99–2.48	0.055
Renal diseases	1.03	0.21–4.99	0.98
Single umbilical artery	0.58	0.10–3.18	0.53
Tobacco use	1.56	0.35–7.05	0.56
Uterine malformation	1.26	0.27–5.78	0.77

*CI, confidence interval; GDM, gestational diabetes mellitus; ICP, intrahepatic cholestasis of pregnancy; OR, odds risk; SE, standard error; SGA, small for gestational age*.

* *, Adjusted for maternal age, socioeconomic status, race/ethnicity, singleton or not and Parity*.

† *, P value from multivariate binary logistic regression analysis*.

## Discussion

We collected population-based, case-control, including risk factors, demographic and clinical characteristics, and outcomes, for full-term SGA infants born to mothers with GDM in China. To our knowledge, this is the first study to report that SGA infants born to mothers with GDM had more adverse perinatal outcomes than SGA infants born to mothers without GDM, the girls of these infants had accelerated CUG in the first year of life.

It has long been understood that the pathogenesis of SGA infants includes both genetic and environmental components (13). The recurrence risk among siblings is defined as the rate of disease frequency among siblings and is widely used to measure the shared genetic contributions (14–16). The rate of siblings who were SGA among SGA infants born to mothers with GDM was 2-fold higher than that among SGA infants born to mothers without GDM in our study, suggesting that genetic factors may be the primary etiology of SGA infants born to mothers with GDM.

All environmental components are listed in Table 1; multiple birth, placental insufficiency, poor nutrition and hypertensive disorders were found to be common environmental causes for SGA infants; but these risk factors for SGA infants born to mothers with GDM should be derived from multivariable logistic regression analysis by comparing with appropriate-for-gestational-age (AGA) infants born to mothers with GDM (8). However, some interesting phenomena can be found in our study. First, weight gain during gestation was significantly lower in SGA mothers with GDM compared to SGA mothers without GDM, so weight gain during gestation may be a causative factor for these infants. Previous studies also suggested that excessive glucose control among women with GDM may decrease fetal growth (17–21). Therefore, clinicians should pay more attention to women with GDM who have inappropriate weight gain during pregnancy. Second, the rate of placental insufficiency among SGA mothers with GDM was significantly lower than that among SGA mothers without GDM (6.0 vs. 9.5%) in our study; this result suggested that placental insufficiency was not the most common cause of these infants. Third, Pearson's correlation analysis showed that prepregnancy BMI and prepartum period BMI were correlated with the birth weight of SGA infants; previous studies also demonstrated that prepregnancy BMI could be used as a predictor of SGA (17–19).

Our study presented novel information about perinatal complications of SGA infants born to mothers with GDM, which had not been reported. In our study, the risks of low 1- and 5-min Apgar scores (≤3) (3.4 and 1.0%), obstetric trauma (2.1%), hypoglycemia (7.6%), HIE (1.0%), pulmonary hemorrhage (1.0%), and early thrombocytopenia (2.1%) were higher in SGA infants born to mothers with GDM than in SGA infants born to mothers without GDM. A possible association between GDM and birth defects has been reported in some studies (22–24), but this association was not found in our study.

Ninety percent of SGA children will experience CUG in the first 2 years of life, and these infants are likely to have excess central fat and abnormal adipocyte function as well as endocrine system disturbances (25). Recent studies showed that SGA infants and infants born to mothers with GDM who had accelerated CUG in the first 12 months of life had a higher risk of cardiovascular disease and type 2 diabetes in adulthood (26–29). SGA girls born to mothers with GDM had more rapid CUG (24.9 vs.16.0%) in the first year of life than SGA infants born to mothers without GDM in our study, but further large-scale long-term follow-up studies are needed to determine the CVD and type 2 diabetes morbidity in later life. Some studies also showed that SGA infants with CUG and infants born to mothers with GDM had higher risks of poor neurological and cognitive outcomes at school age (30, 31). Therefore, it is important to monitor childhood weight, height and head circumference regularly for SGA infants born to mothers with GDM because these measurements might reveal the need for intervention strategies to reduce their later CVD risk and alleviate their poor neurodevelopmental outcomes.

One of the important strengths of our work is its large sample size of SGA infants born to mothers without GDM. Additionally, this study is an observational case-control study. To the best of our knowledge, this is the first study to report the risk factors, demographic and clinical characteristics and outcomes of full-term SGA infants born to mothers with GDM *via* a population-based case-control study. Our findings are also helpful for clinicians to design more accurate postnatal care programs and follow-up programs for SGA infants born to mothers with GDM.

This study has several limitations. First, the number of GDM mothers who were given drug therapy may be small, the blood glucose of 99.2% GDM women could be controlled through dietary therapy; while in the literature it ranged from 3 to 15%; but these studies showed that there were no significant difference in maternal and neonatal outcomes between diet therapy group and insulin therapy group (32, 33). Second, the duration of follow-up was too short, and too few tools were available to assess the trajectory of the CUG of SGA infants in our study; previous studies suggested that accelerated growth in the first 12 months of life may confer an increased risk of CVD and type 2 diabetes up to the age of 18 years (34, 35). Therefore, long-term, large-scale studies are needed to determine the incidence of CVD and diabetes in SGA infants born to mothers with GDM.

### Conclusions

In conclusion, the incidence of full-term SGA infants born to mothers with GDM was 1 in 155 live births in China. Genetic factors may be an etiology of SGA infants born to mothers with GDM, and other risk factors for these infants should be derived from multivariable logistic regression analysis via comparisons with AGA infants born to mothers with GDM. Compared with SGA infants born to mothers without GDM, SGA infants born to mothers with GDM have a higher risk of perinatal complications (including low 1-min Apgar score, hypoglycemia, hypoxic-ischemic encephalopathy, pulmonary and gastrointestinal hemorrhage, early thrombocytopenia); the girls of these infants had accelerated CUG in the first year of life.

## Data Availability Statement

The original contributions presented in the study are included in the article/supplementary material, further inquiries can be directed to the corresponding author.

## Author Contributions

JC, HX, YY, and WZ designed the study, designed the data collection instruments, collected data, carried out the initial analyses, drafted the initial manuscript, and created the tables and figures. YT, XY, and ZZ conceptualized and designed the study, coordinated and supervised data collection, and helped draft the initial manuscript. WL, JY, and LH designed the data collection instruments, collected data, and conducted the initial analyses. XL conceptualized and designed the work, helped draft the initial manuscript, and provided feedback about the study design. All authors contributed to the manuscript's critical revision and read and approved the submitted version.

## Funding

This article was supported by Science and Technology Planning Project of Guangdong Province (2016A020215177), China.

## Conflict of Interest

The authors declare that the research was conducted in the absence of any commercial or financial relationships that could be construed as a potential conflict of interest.

## Publisher's Note

All claims expressed in this article are solely those of the authors and do not necessarily represent those of their affiliated organizations, or those of the publisher, the editors and the reviewers. Any product that may be evaluated in this article, or claim that may be made by its manufacturer, is not guaranteed or endorsed by the publisher.

## Abbreviations

AGA: appropriate-for-gestational-age
BMI: body mass index
BW: birth weights
CI: confidence interval
CUG: catch-up growth
GDM: gestational diabetes mellitus
HIE: hypoxic-ischemic encephalopathy
ICH: Intracranial hemorrhage
NEC: Necrotizing enterocolitis
NRDS: neonatal respiratory distress syndrome
PI: ponderal index
RR: relative risk
SD: standard deviations
SGA: small for gestational age.

## References

[1] Lee KW Ching SM Ramachandran V Yee A Hoo FK Chia YC. Prevalence and risk factors of gestational diabetes mellitus in Asia: a systematic review and meta-analysis. BMC Pregnancy Childbirth. (2018) 18:494. 10.1186/s12884-018-2131-4 30547769 PMC6295048

[2] Patterson AG Corcoy R Balsells M Altirriba O Adelantado JM Cabero L. In pregnancies with gestational diabetes mellitus and intensive therapy, perinatal outcome is worse in small-for-gestational-age newborns. Am J Obstet Gynecol. (1998)179:481–5. 10.1016/S0002-9378(98)70383-7 9731857

[3] von Beckerath AK Kollmann M Rotky-Fast C Karpf E Lang U Klaritsch P.Perinatal complications and long-term neurodevelopmental outcome of infants with intrauterine growth restriction. Am J Obstet Gynecol. (2013) 208:130.e1–6. 10.1016/j.ajog.2012.11.014 23159694

[4] Beukers F Aarnoudse-Moens CSH van Weissenbruch MM Ganzevoort W van Goudoever JB van Wassenaer-Leemhuis AG. Fetal growth restriction with brain sparing: neurocognitive and behavioral outcomes at 12 years of age. J Pediatr. (2017)188:103–9.e2. 10.1016/j.jpeds.2017.06.003 28693788

[5] Esakoff TF Guillet A Caughey AB. Does small for gestational age worsen outcomes in gestational diabetics? J Matern Fetal Neonatal Med. (2016) 30:1–4. 10.1080/14767058.2016.1193142 27269646

[6] Borqono CA Hamiton JK Ye C Connelly PW Sermer M Zinman B. Determinants of insulin resistance in infants at age 1 year: impact of gestational diabetes mellitus. Diabetes Care. (2012) 35:1795-7. 10.2337/dc12-0173 22699283 PMC3402255

[7] Abokaf H Shoham-Vardi I Sergienko R Landau D Sheiner E. In utero exposure to gestational diabetes mellitus and long term endocrine morbidity of the offspring. Diabetes Res Clin Pract. (2018) 144:231–5. 10.1016/j.diabres.2018.09.003 30213770

[8] Saleem T Sajjad N Fatima S Habib N. Ali SR Qadir M. Intrauterine growth retardation—small events, big consequences. Ital J Pediatr. (2011) 37:41. 10.1186/1824-7288-37-41 21899747 PMC3177763

[9] Kerkhof GF Willemsen RH Leunissen RW Breukhoven PE. Hokken-Koelega AC.Health profile of young adults born preterm: negative effects of rapid weight gain in early life. J Clin Endocrinol Metab. (2012) 97:4498–506. 10.1210/jc.2012-1716 22993033

[10] Leunissen RW Kerkhof GF Stijnen T Hokken-Koelega A. Timing and tempo of first-year rapid growth in relation to cardiovascular and metabolic risk profile in early adulthood. JAMA. (2009) 01:2234–42. 10.1001/jama.2009.761 19491185

[11] International Association of Diabetes and Pregnancy Study Groups Consensus Panel. International Association of Diabetes and Pregnancy Study Groups recommendations on the diagnosis and classification of hyperglycemia in pregnancy. Diabetes Care. (2010) 33:676–82. 10.2337/dc09-1848 20190296 PMC2827530

[12] MOH of the PRC. The growth reference standard of Chinese children under 7 years (in Chinese). The Ministry of Health of the People's Republic of China. (2020).

[13] Bocca-Tjeertes I Bos A Kerstjens J Winter AD Reijneveld S. Symmetrical and asymmetrical growth restriction in preterm-born children. Pediatrics. (2014) 133:e650. 10.1542/peds.2013-1739 24488742

[14] Villanueva R Greenberg DA Davies TF Tomer Y. Sibling recurrence risk in autoimmune thyroid disease. Thyroid. (2003)13:761–4. 10.1089/105072503768499653 14558919

[15] Palmer N Beam A Agniel D Eran A Manrai A. Spettell C. Association of sex with recurrence of autism spectrum disorder among siblings. JAMA pediatr. (2017) 171:1107–12. 10.1001/jamapediatrics.2017.2832 28973142 PMC5710368

[16] Ozonoff SJ Young GS Carter AS Messinger D Yirmiya N Zwaigenbaum L. Recurrence risk for autism spectrum disorders: A baby siblings research consortium study. Pediatrics. (2011)128:e488–95. 10.1542/peds.2010-2825 21844053 PMC3164092

[17] Goldstein RF Abell SK Ranasinha S Misso M Boyle JA. Black MH. Association of gestational weight gain with maternal and infant outcomes: a systematic review and meta-analysis. JAMA. (2017) 27:2207–25. 10.1001/jama.2017.3635 28586887 PMC5815056

[18] Diemert A Lezius S Pagenkemper M Hansen G Drozdowska A Hecher K. Maternal nutrition, inadequate gestational weight gain and birth weight: results from a prospective birth cohort. BMC Pregnancy Childbirth. (2016) 16:224. 10.1186/s12884-016-1012-y 27528213 PMC4986204

[19] Gavard JA Artal R. The association of gestational weight gain with birth weight in obese pregnant women by obesity class and diabetic status: a population-based historical cohort study. Matern Child Health J. (2014) 18:1038–47. 10.1007/s10995-013-1356-0 24077985

[20] Kurtzhals LL Norgaard SK Secher AL Nichum VL Ronneby H Tabor A. The impact of restricted gestational weight gain by dietary intervention on fetal growth in women with gestational diabetes mellitus. Diabetologia. (2018) 61:2528–38. 10.1007/s00125-018-4736-6 30255376

[21] Barquiel B Herranz L Martínez-Sánchez N Montes C Hillman N Bartha JL. Increased risk of neonatal complications or death among neonates born small for gestational age to mothers with gestational diabetes. Diabetes Res Clin Pract. (2020) 159:107971. 10.1016/j.diabres.2019.107971 31805352

[22] Parnell AS Correa A Reece EA. Pre-pregnancy obesity as a modifier of gestational diabetes and birth defects associations: a systematic review. Matern Child Health J. (2017) 21:1105–20. 10.1007/s10995-016-2209-4 28120287

[23] Correa A Gilboa SM Besser LM Botto LD Moore CA Hobbs CA. Diabetes mellitus and birth defects. Am J Obstet Gynecol. (2008) 199:237.e1–9. 10.1016/j.ajog.2008.06.028 18674752 PMC4916956

[24] Shnorhavorian M Bittner R Wright JL Schwartz SM. Maternal risk factors for congenital urinary anomalies: results of a population-based case-control study. Urology. (2011) 78:1156–61. 10.1016/j.urology.2011.04.022 22054394

[25] Singhal A. Long-term adverse effects of early growth acceleration or catch-up growth. Ann Nutr Metab. (2017) 70:236–40. 10.1159/000464302 28301849

[26] Shi H Yang X Wu D Wang X Li T Liu H. Insights into infancy weight gain patterns for term small-for-gestational-age babies. Nutr J. (2018) 17:97. 10.1186/s12937-018-0397-z 30373572 PMC6206641

[27] Kelishadi R Haghdoost AA Jamshidi F Aliramezany M Moosazadeh M. Low birthweight or rapid catch-up growth: which is more associated with cardiovascular disease and its risk factors in later life? A systematic review and cryptanalysis. Paediatr Int Child Health. (2015) 35:110–23. 10.1179/2046905514Y.0000000136 25034799

[28] Singhal A Cole TJ Fewtrell M Kennedy K Stephenson T Elias-Jones A. Promotion of faster weight gain in infants born small for gestational age: is there an adverse effect on later blood pressure? Circulation. (2007) 115:213–20. 10.1161/CIRCULATIONAHA.106.617811 17179023

[29] Chomtho S Wells JC Williams JE Davies PS Lucas A Fewtrell MS. Infant growth and later body composition: evidence from the 4-component model. Am J Clin Nutr. (2008) 87:1776–84. 10.1093/ajcn/87.6.1776 18541568

[30] Vollmer B. Edmonds CJ. School age neurological and cognitive outcomes of fetal growth retardation or small for gestational age birth weight. Front Endocrinol (Lausanne). (2019) 10:186. 10.3389/fendo.2019.00186 30984109 PMC6447606

[31] Lee KW Ching SM Hoo FK Ramachandran V Chong SC. Tusimin M. Neonatal outcomes and its association among gestational diabetes mellitus with and without depression, anxiety and stress symptoms in Malaysia: A cross-sectional study. Midwifery. (2020) 81:102586. 10.1016/j.midw.2019.102586 31830674

[32] Martis R Crowther CA Shepherd E Alsweiler J Downie MR Brown J. Treatments for women with gestational diabetes mellitus: an overview of Cochrane systematic reviews. Cochrane Database Syst Rev. (2018) 8:CD012327. 10.1002/14651858.CD012327.pub2 30103263 PMC6513179

[33] Silva AL Amaral AR Oliveira DS Martins L Silva MR Silva JC. Neonatal outcomes according to different therapies for gestational diabetes mellitus. J Pediatr (Rio J). (2017) 93:87–93. 10.1016/j.jped.2016.04.004 27371343

[34] Fabricius-Bjerre S Jensen RB Færch K Larsen T Mølgaard C Michaelsen KF. Impact of birth weight and early infant weight gain on insulin resistance and associated cardiovascular risk factors in adolescence. PLoS ONE. (2011) 6:e20595. 10.1371/journal.pone.0020595 21655104 PMC3107215

[35] Neimark E Wainstock T Sheiner E Fischer L Pariente G. Long-term cardiovascular hospitalizations of small for gestational age (SGA) offspring born to women with and without gestational diabetes mellitus (GDM) . Gynecol Endocrinol. (2019) 35:518–24. 10.1080/09513590.2018.1541233 30626227

